# Heat-killed *Lactobacillus acidophilus* suppresses SARS-CoV-2 infection in the human intestinal epithelial cell line Caco-2

**DOI:** 10.3389/fcimb.2025.1556344

**Published:** 2025-07-31

**Authors:** Kazuhide Takada, Quang Duy Trinh, Yoshinori Takeda, Noriko M. Tsuji, Satoshi Hayakawa, Shihoko Komine-Aizawa

**Affiliations:** ^1^ Division of Microbiology, Department of Pathology and Microbiology, Nihon University School of Medicine, Tokyo, Japan; ^2^ Division of Immune Homeostasis, Department of Pathology and Microbiology, Nihon University School of Medicine, Tokyo, Japan; ^3^ Department of Obstetrics and Gynecology, Nara Medical University, Nara, Japan; ^4^ Department of Food Science, Jumonji University, Saitama, Japan

**Keywords:** COVID-19, long COVID, probiotics, gut, lactobacillus, SARS-CoV-2

## Abstract

**Background:**

The gastrointestinal (GI) tract is suspected to be a possible source for the systemic spread of severe acute respiratory syndrome coronavirus 2 (SARS-CoV-2), as well as a reservoir of long coronavirus disease (COVID). Thus, the mucosal epithelial tissue of the colon is a potential target for probiotics to help control SARS-CoV-2 infection. Recently, the effect of live probiotics on COVID-19 has been evaluated. However, live probiotics have certain risks, including the transmission of antibiotic-resistant genes, disturbance of gut colonization in infants, and systemic infections induced by translocation. Therefore, there is growing interest in nonviable microorganisms, particularly heat-killed probiotic bacteria, to mitigate these risks.

**Methods:**

This study evaluated the antiviral properties of heat-killed *Lactobacillus acidophilus* (HK-LA) in the Caco-2 cell line. Caco-2 cells were infected by SARS-CoV-2 with or without 24-hour pretreatment of HK-LA and the presence of HK-LA during infection.

**Results:**

RT-qPCR analysis showed that HK-LA treatment significantly reduced SARS-CoV-2 genome copies by approximately 30%. Similarly, flow cytometry revealed a roughly 30% decrease in SARS-CoV-2 spike-positive Caco-2 cells following HK-LA treatment. Additionally, ELISA demonstrated a significant increase in IFN-λ2 secretion induced by HK-LA.

**Discussion:**

HK-LA reduces viral infection in Caco-2 cells with an increase in IFN-λ2 secretion. Therefore, heat-killed lactobacilli could potentially reduce SARS-CoV-2 infection in the GI tract, suggesting a possible clinical application.

## 1 Introduction

The World Health Organization (WHO) has formally revoked the designation of coronavirus disease 2019 (COVID-19) as a “global health emergency”; however, it continues to present a substantial global health threat (Wise, 2023). The alpha and delta strains prevalent at the start of the epidemic have been replaced by omicron strains and their sub-strains over the past two years. Additionally, an increase in the number of vaccinated and previously infected individuals has led to the establishment of herd immunity (Suryawanshi and Biswas, 2023). Consequently, the number of patients experiencing fatal outcomes has significantly reduced. However, there is still a notable occurrence of severe cases, and the primary challenge lies in managing long COVID caused by persistent infection. Many countries have experienced a decline in the number of surplus vaccines due to the cessation of public support, which was prompted by economic crises in Japan and other countries. Moreover, the use of antiviral medications is limited because of their high cost and potential problems with drug interactions (Wise, 2023; Neris Almeida Viana et al., 2024).

Interestingly, probiotics have been demonstrated to be effective in treating COVID-19 in this particular situation (Neris Almeida Viana et al., 2024). Although their exact molecular action mechanism is not well understood, probiotics are believed to enhance the host antiviral immune response and regulate the gut microbiota, which is thought to alleviate COVID-19 (Nguyen et al., 2022). Furthermore, it has been proposed that the gastrointestinal (GI) tract may act as a possible pathway for the spread of the virus within an infected individual and as a reservoir of long COVID-19 (Neurath et al., 2021). Therefore, it is essential to develop efficient probiotics to control SARS-CoV-2 infection in the GI tract by eradicating SARS-CoV-2-infected cells. Several studies have utilized live bacteria for this purpose. However, they may pose certain risks, including the transmission of antibiotic-resistant genes, disturbance of gut colonization in infants, and systemic infections induced by translocation. These risks are particularly significant in susceptible people and the pediatric population (Piqué et al., 2019). Therefore, there is growing interest in nonviable microorganisms, particularly heat-killed probiotic bacteria, to mitigate these risks (Piqué et al., 2019). In this study, we assessed the effect of heat-killed *Lactobacillus acidophilus* (HK-LA) on the infection of SARS-CoV-2 in Caco-2, a human immortalized intestinal epithelial cell line.

## 2 Methods

### 2.1 Cell culture

Minimum essential medium (MEM) (Gibco, Carlsbad, CA, USA) with the addition of fetal bovine serum (FBS) (20%) was used to culture Caco-2 cells. Antibiotics (100 U/mL of penicillin and streptomycin) and 100 mM nonessential amino acids (NEAA) (Gibco, Carlsbad, CA, USA) were also added to the medium. Cultivation was carried out in a CO_2_ incubator (5%: Hirasawa, Tokyo, Japan) at 37°C.

### 2.2 Bacterial culture and HK-LA

*L. acidophilus* strain JCM2124 was purchased from RIKEN BioResource Research Center (Ibaraki, Japan). *L. acidophilus* (JCM2124) was grown in de Man, Rogosa, and Sharpe (MRS) broth (Becton, Dickinson and Company, Sparks, MD, USA) at 37°C without shaking. Optical turbidity at 600 nm was measured by using a spectrophotometer (Amersham Pharmacia Biotech, Cardiff, UK) to monitor bacterial growth. Bacterial numbers were evaluated as colony-forming units (CFUs) (Hayashida et al., 2022).

HK-LA was prepared as described previously (Saito et al., 2020) with a few modifications. In short, the 10 ml of *L. acidophilus* culture (10^7^ CFU/ml) were killed at 95°C for 10 min, after which centrifugation (5000 ×*g*, 10 min) was performed to precipitate the bacterial cells. After the washing step with 0.9% NaCl, the bacterial pellet was reconstituted in saline solution. Then, the samples were stored at -80°C in an ultra-low temperature freezer (ULT-1390-10-D; Thermo Fisher Scientific, Waltham, MA, USA) until further use.

### 2.3 Viral infection

For the viral infection experiment, HK-LA (multiplicity of infection (MOI) 1:5 or 1:50) or saline (as a vehicle) was added to the Caco-2 cell culture mixture. After 24 h, the cell culture medium was changed to MEM (2% FBS), and HK-LA or saline was added again. The cells were subsequently infected with severe acute respiratory syndrome coronavirus 2 (SARS-CoV-2) (WK-521 strain, provided by the National Institute of Infectious Diseases, Tokyo, Japan) (MOI of 1:0.5) and cultured in a CO_2_ incubator (5%) at 37°C for another 24 h. The viruses were propagated and titrated in VeroE6/TMPRSS2 cells. All infection experiments in this study were conducted in a biosafety level 3 (BSL-3) research zone at the Nihon University School of Medicine with the approval of the institute’s biosafety committee.

### 2.4 RT-qPCR

After 24 h of viral infection, RNA was extracted using the ReliaPrep RNA Cell Miniprep System (Promega, WI, USA). Reverse transcription and amplification of viral RNA were performed with the One Step PrimeScript III RT-qPCR Mix (Takara, Tokyo, Japan) using virus-specific primers and probes (Primer/Probe N2 (2019-nCoV); Takara). Reactions were carried out on a QuantStudio 5 Flex Real-Time PCR System (Life Technologies, Carlsbad, CA, USA).

### 2.5 Flow cytometry

After 24 h of SARS-CoV-2 infection, the cells were washed twice with phosphate-buffered saline (PBS) and then trypsinized. They were subsequently treated with LIVE/DEAD fixable dead cell stains (Thermo Fisher Scientific) to exclude dead cells. Then, the cells were fixed and permeabilized by BD Cytofix/Cytoperm solution (BD Biosciences, Franklin Lakes, NJ, USA). Finally, the cells were stained for the SARS-CoV-2 spike S1 subunit using its antibody (FAB105403G; R&D Systems, Minneapolis, MN, USA). We performed fluorescence-activated cell sorting (FACS) using a FACSVerse flow cytometer (BD Biosciences, Franklin Lakes, NJ, USA). The result of FACS was then evaluated by FlowJo software (BD Biosciences). The gating strategy is shown in **Supplementary Figure S1**.

### 2.6 Western blot

After 24 h of incubation with HK-LA (MOI 1:50), Caco-2 cells were washed with PBS and lysed using cell lysis buffer (Cell Signaling Technology, Danvers, MA, United States). Cell lysates were loaded onto a NuPAGE 4–12% Bis-Tris protein gel (Invitrogen) and separated by electrophoresis. The separated proteins were then transferred onto polyvinylidene fluoride membranes (Invitrogen). Membranes were incubated overnight at 4°C with primary antibodies against Angiotensin-converting enzyme 2 (ACE2) (1:500; Abcam, Cambridge, United Kingdom), Transmembrane protease serine 2 (TMPRSS2) (1:500; Abcam), or α-Tubulin served as the internal control (1:1000; Cell Signaling Technology). Membranes were subsequently incubated with horseradish peroxidase-conjugated secondary antibodies (Cell Signaling Technology) for 30 min at room temperature and visualized using an LAS-4000 Mini image analyzer (Fujifilm, Tokyo, Japan).

### 2.7 ELISA

Cell culture supernatants were collected 24 h after incubation with HK-LA (MOI 1:50). The samples were centrifuged at 8000 × g for 5 minutes to remove cells and debris and subsequently stored at -20°C for later analysis. Protein concentrations of interferon (IFN)-α and IFN-λ2 were measured using ELISA kits (R&D Systems Inc., Minneapolis, MN, USA and RayBiotech, Peachtree Corners, GA, USA, respectively) following the manufacturer’s instructions.

### 2.8 Statistical analysis

For statistical analysis, Statcel4 (OMS Publishing Inc., Tokyo, Japan) was employed. The results were evaluated using Student’s *t*-test or the Tukey–Kramer test. Statistical significance was set at p < 0.05.

## 3 Results

**Figure 1:**
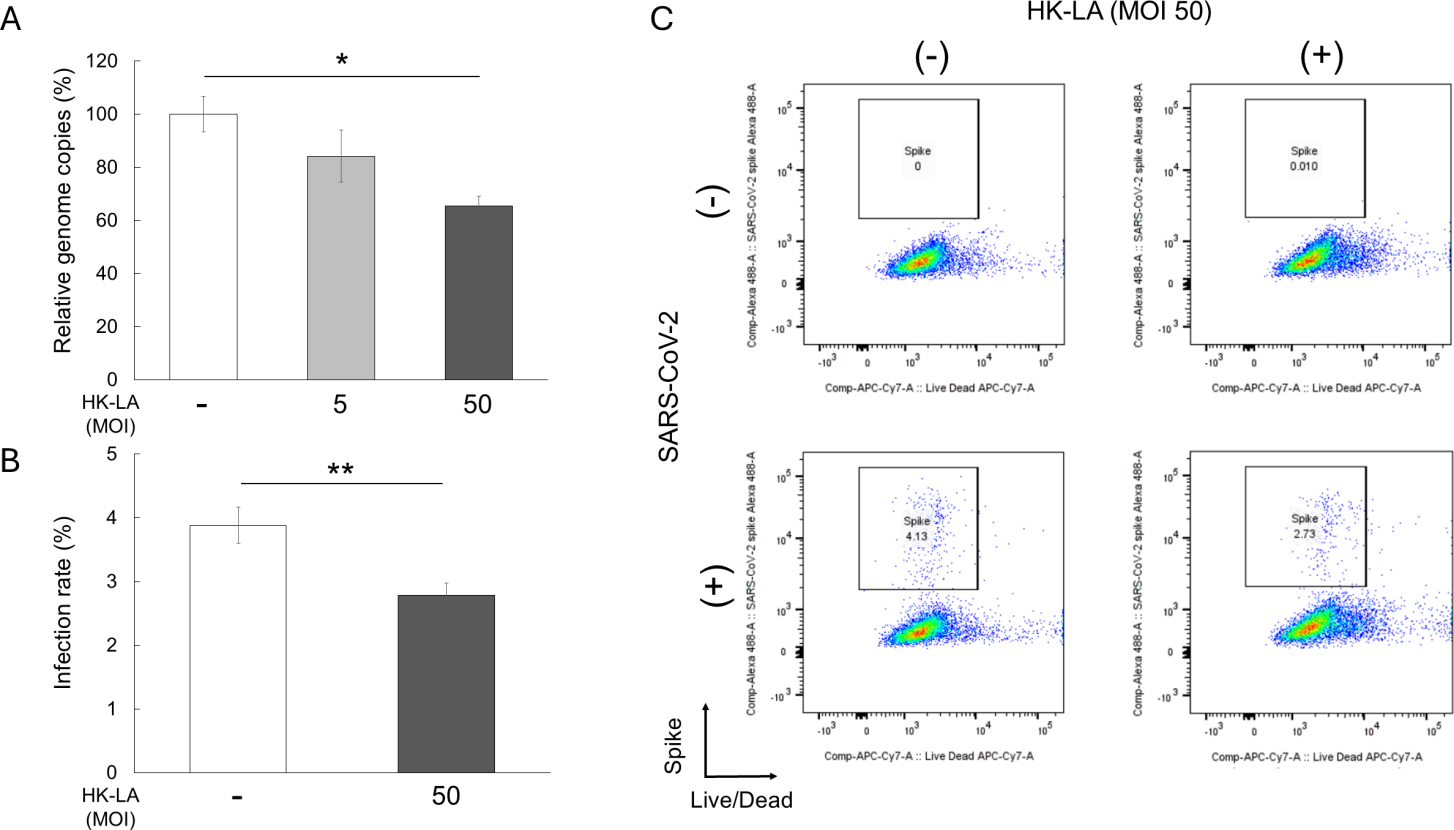
Anti-SARS-CoV-2 effect of heat-killed *Lactobacillus acidophilus* (HK-LA). HK-LA treatment significantly reduced SARS-CoV-2 genome copies by approximately 30%, as confirmed by RT-qPCR **(A)**. HK-LA also significantly reduced the number of Caco-2 cells positive for the SARS-CoV-2 spike S1 subunit after 24 h of SARS-CoV-2 infection **(B, C)**. The infection and reduction rates are approximately 4% and 30%, respectively **(B)**. The data represent the mean ± SEM; *p < 0.05 or **p < 0.01 according to the Tukey–Kramer test **(A)** or Student’s *t*-test **(B)** (n = 3-4).

Caco-2 cells were infected with SARS-CoV-2 following HK-LA treatment. After 24 h, HK-LA (MOI 1:50) significantly reduced viral genome copies by approximately by 30% in a dose-dependent manner (**Figure 1A**). This finding was confirmed by flow cytometry. The HK-LA (MOI 1:50) treatment significantly reduced the number of Caco-2 cells infected with SARS-CoV-2 (**Figures 1B, C**, **Supplementary Figure S2**). The infection rate was approximately 4%, and HK-LA treatment reduced the infection rate by approximately 30% (**Figure 1B**).

**Figure 2:**
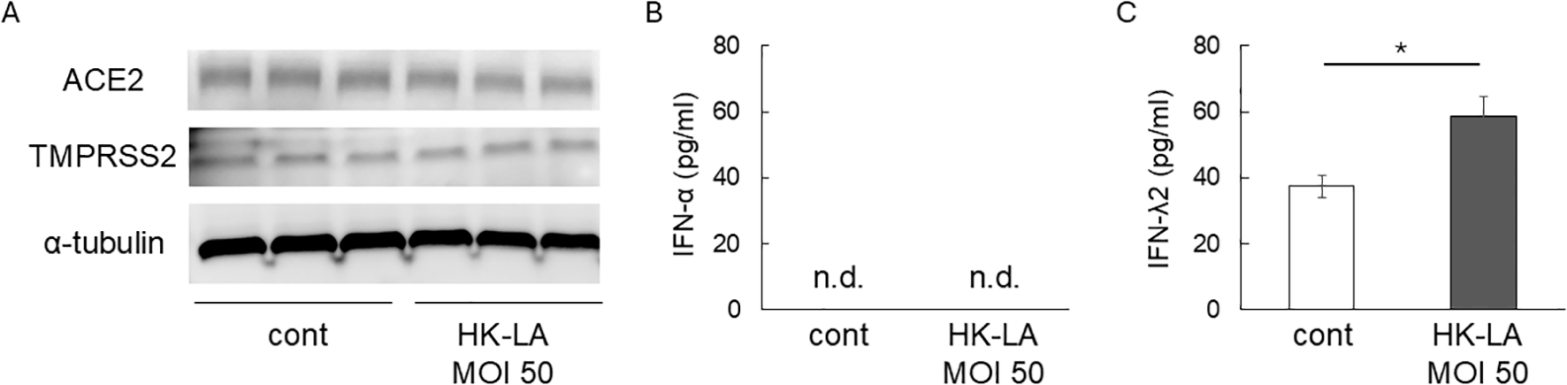
Potential mechanisms of HK-LA against SARS-CoV-2 infection. Western blot analysis showed that 24-h HK-LA treatment did not affect the protein expression of ACE2 or TMPRSS2 in Caco-2 cells **(A)**. HK-LA treatment significantly increased the secretion of IFN-λ2 **(C)**, while IFN-α was not detected in the cell culture supernatant **(B)**. Data represent the mean ± SEM; *p < 0.05 according to Student’s t-test **(C)** (n=6). n.d.; not detected.

Next, we evaluated the effect of HK-LA on viral receptors. HK-LA treatment did not affect the expression of ACE2 and TMPRSS2, which are known SARS-CoV-2 receptors in humans (Hoffmann et al., 2020) (**Figure 2A** We then measured the concentration of Type I and III IFNs, which play protective roles against SARS-CoV-2 infection (Felgenhauer et al., 2020; Vanderheiden et al., 2020). ELISA results showed that Caco-2 cells did not secrete IFN-α even after HK-LA treatment (**Figure 2B**). In contrast, HK-LA treatment significantly increased the secretion of IFN-λ2 (**Figure 2C**).

## 4 Discussion

Growing evidence suggests that live probiotics are effective in combating SARS-CoV-2 infections in the GI tract. The presence of live *Lacticaseibacillus paracasei* (formerly known as *Lactobacillus paracasei*) was shown to effectively suppress SARS-CoV-2 infection and enhance the antiviral immune response of lactoferrin in Caco-2 cells (Salaris et al., 2021). In addition to the risks mentioned above, administering live bacteria poses challenges, such as difficulties in establishing themselves as permanent members of the intestinal flora. Our study demonstrates the effectiveness of nonviable *L. acidophilus* in preventing SARS-CoV-2 infection in a human immortalized gut epithelial cell line, as observed in live bacteria. While the exact processes by which lactobacilli defend against COVID-19 are not well understood, numerous potential mechanisms have been proposed.

We propose that dead lactobacilli might activate local immunomodulatory pathways. Previous studies have suggested that cytokine production by lactobacilli can be controlled via the upregulation of anti-inflammatory reactions (Mahooti et al., 2020; Taufer et al., 2024). In the present study, HK-LA treatment influenced the secretion of IFN-λ2. A previous study demonstrated that IFN-λ treatment provided protective effects against SARS-CoV-2 both *in vitro* and in human-colon-derived organoids (Stanifer et al., 2020). Therefore, the protective effect of HK-LA observed in the present study may involve the IFN-λ signaling pathway. Additionally, it is important to acknowledge the presence of direct antiviral mechanisms, including the intracellular mechanisms, such as the activation of retinoic acid-inducible gene-I (RIG-I) and Toll-like receptor (TLR), which inhibit viral replication (Taufer et al., 2024). Enhancement of the epithelial barrier by lactobacilli (Abramov et al., 2021) on the mucosal surface is also crucial for preventing viral penetration into the GI tract tissue (Wu et al., 2024). In conclusion, HK-LA reduces SARS-CoV-2 infection in the GI tract and holds promise for future therapeutic applications.

## Data Availability

The original contributions presented in the study are included in the article/**Supplementary Material**. Further inquiries can be directed to the corresponding author/s.
