# Supplementary figures and images for: Heat-killed Lactobacillus acidophilus suppresses SARS-CoV-2 infection in the human intestinal epithelial cell line Caco-2
Source: Front Cell Infect Microbiol. 2025 Jul 31;15:1556344. doi: 10.3389/fcimb.2025.1556344 (PMC12350398; doi:10.3389/fcimb.2025.1556344)

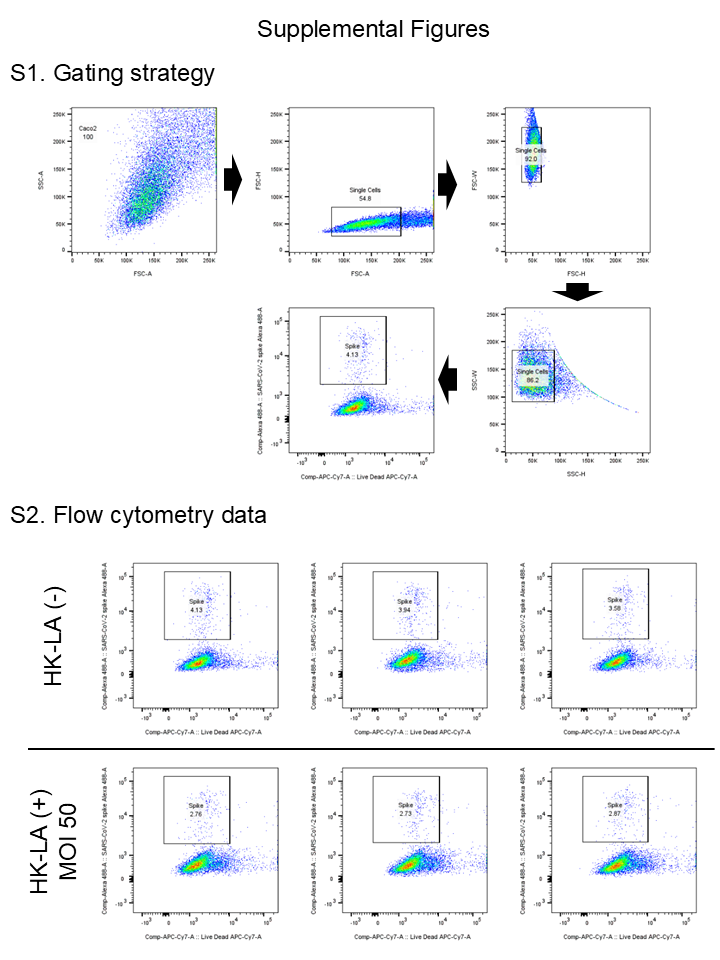

Supplement: Supplementary file 1 [file Image1.tif]
